# Motor Timing in Tourette Syndrome: The Effect of Movement Lateralization and Bimanual Coordination

**DOI:** 10.3389/fneur.2019.00385

**Published:** 2019-04-26

**Authors:** Davide Martino, Andreas Hartmann, Elisa Pelosin, Giovanna Lagravinese, Cecile Delorme, Yulia Worbe, Laura Avanzino

**Affiliations:** ^1^Department of Clinical Neurosciences, University of Calgary and Hotchkiss Brain Institute, Calgary, AB, Canada; ^2^Sorbonne Université, UMR S 1127, CNRS UMR 7225, ICM, Paris, France; ^3^Department of Neurology, Groupe Hospitalier Pitié-Salpêtrière, Assistance Publique-Hôpitaux de Paris, 47-83 boulevard de l'Hôpital, Paris, France; ^4^French National Reference Centre for Gilles de la Tourette Syndrome, Groupe Hospitalier Pitié-Salpêtrière, Paris, France; ^5^Department of Neuroscience, Rehabilitation, Ophthalmology, Genetics and Maternal Child Health, Genoa, Italy; ^6^Ospedale Policlinico San Martino-IRCCS, Genoa, Italy; ^7^Department of Physiology, Saint-Antoine Hospital, Paris, France; ^8^Section of Human Physiology, Department of Experimental Medicine, University of Genoa, Genoa, Italy

**Keywords:** Tourette syndrome, timing, supplementary motor area, motor control, MRI, bimanual

## Abstract

The study of motor timing informs on how temporal information integrates with motor acts. Cortico-basal ganglia and cortico-cerebellar circuits control this integration, whereas transcallosal interhemispheric connectivity modulates finely timed lateralized or bimanual actions. Motor timing abilities are under-explored in Tourette syndrome (TS). We adopted a synchronization-continuation task to investigate motor timing in sequential movements in TS patients. We studied 14 adult TS patients and 19 age-matched healthy volunteers. They were asked to tap in synchrony with a metronome cue (SYNC) and then, when the tone stopped, to keep tapping, maintaining the same rhythm (CONT). We tested both a sub-second and a supra-second inter-stimulus interval between the cues. Subjects randomly performed a single-hand task with the right hand and a bimanual task using both hands simultaneously wearing sensor-engineered gloves. We measured the temporal error and the interval reproduction accuracy index. We also performed MRI-based diffusion tensor imaging and probabilistic tractography of inter-hemispheric corpus callosum (CC) connections between supplementary motor areas (SMA) and the left SMA-putamen fiber tract. TS patients were less accurate than healthy individuals only on the single-hand version of the CONT task when asked to reproduce supra-second time interval. Supra-second time processing improved in TS patients in the bimanual task, with the performance of the right hand on the bimanual version of the CONT task being more accurate than that of the right hand on the single-hand version of the task. We detected a significantly higher fractional anisotropy (FA) in both SMA-SMA callosal and left-sided SMA-putamen fiber tracts in TS patients. In TS patients only, the structural organization of transcallosal connections between the SMAs and of the left SMA-putamen tract was higher when the motor timing accuracy of the right hand on the bimanual version of the task was lower. Abnormal timing performance for supra-second time processing is suggestive of a defective network inter-connecting the striatum, the dorsolateral prefrontal cortex and the SMA. An increase in accuracy on the bimanual version of the CONT task may be the result of compensatory processes linked to self-regulation of motor control, as witnessed by plastic rearrangement of inter-hemispheric and cortical-subcortical fiber tracts.

## Introduction

The study of motor timing explores the processing of information on temporal durations during the preparation and execution of motor actions (1–4). Motor timing is a key functional domain influencing the efficiency and the appropriateness to the context of any motor output. Both cortico-basal ganglia and cerebellar networks are involved in this neural computation (5–9), and represent also the pivotal circuitries in the generation of abnormal movements. Furthermore, inter-hemispheric connectivity, primarily supported by callosal fibers, integrates temporal information for finely timed lateralized or bimanual acts (10, 11). Despite the relevance of time processing to the organization of motor control and the overlap between neural networks of motor timing and the neural substrate of tic generation, data on motor timing abilities in patients with Tourette syndrome (TS) are scarce (12). Similar to other uncontrollable, abnormal movements like chorea and dystonia, the temporal pattern of tic expression appears to have a random, albeit repetitive, character, which is clearly different from the appropriately contextualized timing of voluntary, goal-directed movements. Unlike other abnormal movements, on the other hand, tics are characterized by the subject's ability to inhibit them on demand, albeit with variable efficiency. This ability implies a time-sensitive motor control that aims to interrupt the chronological succession of premonitory urge and tic; the timing aspect of this inhibitory effort is relevant to the proficient application of strategies to diminish tics used in behavioral treatment approaches, e.g., habit reversal therapy or exposure response prevention. Moreover, anecdotal and science literature (13) reports an association of TS patients with altered sense and perception of time, including speed of motor actions. Therefore, motor timing performance in patients with tics represents a topic deserving greater attention and investigation.

Motor timing can be directly evaluated by performing explicit timing tasks, in which subjects make explicit use of temporal information (e.g., estimates of the duration of stimuli or intervals between stimuli) to represent precise temporal durations through a motor action (4). One of the most commonly adopted explicit timing tasks is the synchronization-continuation paradigm, which involves the time-controlled execution of sequential movements. This paradigm consists in: (i) a synchronization –or externally triggered- phase, in which subjects are asked to tap in synchrony with a train of tones separated by a constant inter-stimulus interval (ISI), and (ii) a continuation –or internally triggered- phase, in which subjects are requested to continue tapping at the previous rate in the absence of the auditory cue. The difference in external/internal drive between these two phases is consistent with differences in their neural substrate, with the continuation phase being selectively associated with the activation of a network connecting the supplementary motor area, the left caudal putamen and the left ventrolateral thalamus (14).

In this study, we apply the synchronization-continuation paradigm to adults with TS and age-matched healthy volunteers, and explore the relationship between performance on this task and structural connectivity of putatively relevant neural networks. Furthermore, since performance on the synchronization-continuation test is largely dependent on the duration of the inter-stimulus interval (ISI), we tested both a sub-second (metronome rate: 2 Hz, ISI: 500 ms) and a supra-second (metronome rate: 0.5 Hz, ISI: 2,000 ms) inter-stimulus interval between the cues. Importantly, processing of different interval durations in explicit timing tasks may also have a neural correlate, as cerebellar networks appear to be specifically involved in the processing of sub-second intervals when synchronization to an external rhythm is required. Finally, in the version of the synchronization-continuation paradigm adopted herein, subjects performed both a single-hand task with the right hand and a bimanual task using both hands simultaneously. This distinction is also relevant to TS, as we have previously shown in the same sample, that adult TS patients exhibit an abnormal ability to lateralize finger movements in sequential tasks, with increased accuracy when the task is performed bimanually (15). In this previous study, we also documented the loss of a physiological association between lateralization ability and transcallosal connectivity of motor cortical regions. However, the timing aspect of this motor lateralization pattern has never been investigated.

The first aim of our study was to explore the presence of differences in performance on this explicit timing paradigm between TS patients and healthy subjects, and whether these differences are specific for sub-second or supra-second time processing, as well as for the externally triggered (SYNC) mode or the internally triggered (CONT) mode on the single hand task. Given the established relationship between tic generation and the sensorimotor loop of the cortico-basal ganglia circuitry, we anticipate that TS patients with tics persisting in adulthood will manifest decreased accuracy on the explicit timing task explored herein. In particular, given the hypothesized specific role of the sensorimotor loop of the cortico-basal ganglia in sustaining explicit timing activities related to previously learned time durations (16), we anticipate: (i) a greater loss of accuracy in TS patients on the continuation phase for longer duration intervals; (ii) a negative correlation between timing accuracy and tic severity; (iii) a positive correlation between the structural organization of the fiber tracts connecting supplementary motor area and putamen of the left hemisphere and timing accuracy of the right hand in TS patients.

The second aim of our study was to evaluate whether single hand vs. bimanual mode of execution of the task influences explicit motor timing accuracy. In line with our previous findings showing abnormal ability to lateralize finger movements (15), we anticipate decreased accuracy on the explicit timing task when this is performed with a single hand compared to bimanual mode. Moreover, based on our previous work, we hypothesize a lack of association between the ability to lateralize motor performance and the structural organization of the callosal tracts interconnecting the supplementary motor area of the two hemispheres.

## Materials and Methods

### Participants

Patients were recruited from the National Reference Center for Gilles de la Tourette syndrome at the Pitié-Salpêtrière Hospital in Paris, France. Fourteen patients with TS (8 males; mean age 32.9 ± 9.9 SD years) participated in the study. The sample of TS patients recruited in the present study is the same explored in our previous work (15). Inclusion criteria for patients were: (i) age >18 years; (ii) having a confirmed diagnosis of TS according to the Diagnostic and Statistical Manual of Mental Disorders-5 (DSM-5) criteria (17). We applied the following exclusion criteria: (i) co-occurrence of Axis I psychiatric disorders, established by the Mini International Neuropsychiatry Inventory (18), with the exception of obsessive-compulsive disorder; co-occurrence of autistic spectrum disorder, substance abuse aside from nicotine, current major depressive episode, current or past diagnosis of psychotic disorder; patients with a current or past diagnosis of ADHD, as per DSM-5 criteria, were also excluded; (ii) any neurologic disorder other than tics; (iii) visual or hearing impairment; (iv) severe orthopedic problems of the upper limb. We recruited 19 age- and sex-matched healthy subjects (HC, 10 males; mean age 31.8 ± 5.1 years) as control subjects from hospital staff or patients' spouses or friends. Exclusion criteria were the same as for TS patients, plus (i) a personal history of tics, and (ii) any concomitant treatment except for oral contraceptives. All participants were right-handed; we confirmed right hand dominance using the Edinburgh Handedness Inventory (19).

Table 1 reports demographic and clinical information for TS patients. Three TS patients were on treatment with antipsychotic drugs (aripiprazole, risperidone, pimozide), one with citalopram, and one with clonazepam; in each of these patients, the medication dose had been stable for at least 4 weeks. Tic severity was assessed using the Yale Global Tic Severity Scale (YGTSS), 0–50 total tic severity score (20). The local ethics committee (Pitie -Salpêtrière Hospital) approved the study and every participant gave informed written consent for participation. The Ethics committee project' number is INSERM C11-34, CPP 97/12.

**Table 1:** Main demographic and clinical data of patients with Tourette syndrome.

**Patient**	**Age (years)**	**Sex**	**Age at onset (years)**	**Yale global tic severity scale (severity subscore/50)**	**Yale global tic severity scale (total score/100)**	**Presence of obsessive-compulsive symptoms**	**Current pharmacological treatment**
1	42	F	6	10	20	OCBs	Clonazepam
2	29	F	6	12	12	No	Aripiprazole
3	38	M	7	18	38	OCD	Citalopram
4	24	M	6	14	14	No	None
5	30	M	8	12	32	OCBs	None
6	33	M	6	8	8	No	None
7	22	M	10	14	24	No	Risperidone
8	24	F	6	22	32	No	None
9	30	F	7	19	29	No	None
10	33	M	6	27	47	No	Pimozide
11	40	F	8	22	32	No	None
12	60	F	5	15	35	No	None
13	24	M	6	14	24	No	None
14	32	M	9	29	37	No	None

*OCBs, obsessive-compulsive behavior; OCD, obsessive-compulsive disorder*.

### Motor Studies

**Figure 1:**
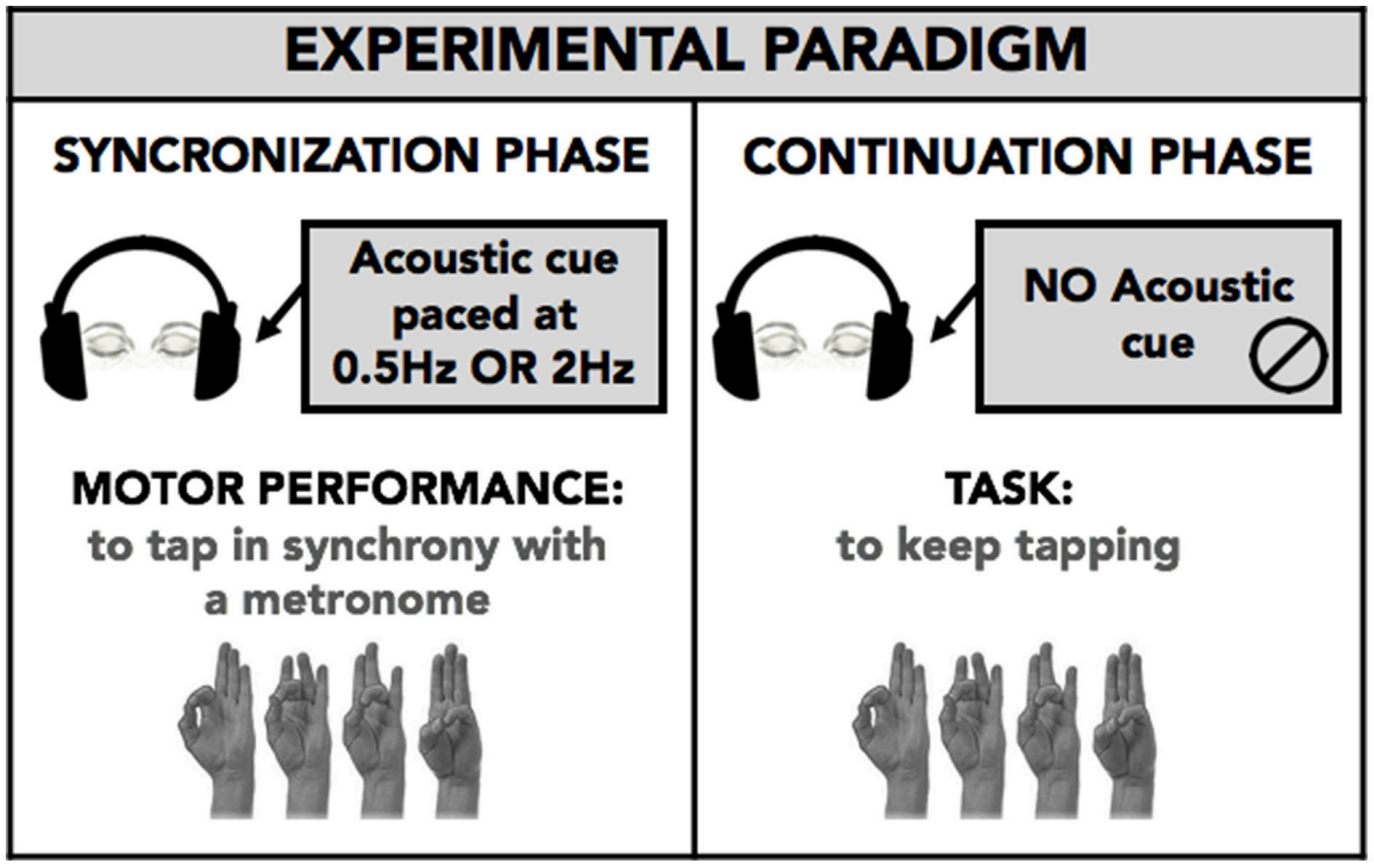
Experimental paradigm. Subject performed with eyes closed a finger opposition movement sequence (opposition of thumb to index, medium, ring, and little fingers) in a synchronization-continuation task. Subjects were requested to tap in synchrony with a metronome cue (SYNC) and then, when the tone stopped, to tap the fingers in a sequential order, trying to maintain the same rhythm as accurately as possible (CONT-EXE). The acoustic cue was set at 0.5 and 2 Hz, to explore timing performance within a supra-second (0.5 Hz, 2,000 ms) and sub-second (2 Hz, 500 ms) time interval. The task was executed with right hand only (single hand task; RH-S) or both the right (RH-B) and left (LH-B) hands simultaneously (bimanual task). The analysis was planned to study for movement lateralization and bimanual coordination.

All subjects performed the motor task. Subjects sat in a comfortable chair in a quiet and darkened room. They wore a sensor-engineered glove (eTT, Genova, Italy) on both their hands. We acquired data at 1 KHz. We chose an eyes closed paradigm to avoid possible confounding effects due to the integration of acoustic and visual information. Subjects were demonstrated the finger sequence task (opposition of thumb to index, medium, ring, and little fingers) only once; subsequently, they were asked to perform the task keeping their eyes closed. The task consisted of performing the finger tapping sequential task in synchrony with a metronome cue (SYNC), and subsequently, when the tone stopped, to continue the same task trying to maintain the same rhythm as accurately as possible (CONT). Each phase (SYNC and CONT) lasted 45 s. Subjects performed two blocks in random order with a different metronome pace (2 Hz, i.e., time interval between two successive metronome cues: 500 ms; 0.5 Hz, i.e., time interval: 2,000 ms). The metronome pace values were chosen in order to have one sub-second time interval (500 ms) and one supra-second time interval (2,000 ms) between two successive auditory stimuli to be reproduced in the CONT task. Subjects randomly performed a single-hand task with the right hand and a bimanual task using both hands simultaneously. Figure 1 summarizes the experimental protocol.

We processed data using a customized software (GAS, eTT, Genoa, Italy) that extracts the duration of the time interval between two successive finger contacts (in ms). In the CONT task, this interval corresponded to the time interval reproduced by the subjects. Performance on the tasks was analyzed by measuring the temporal error and the interval reproduction accuracy index (1). The temporal error corresponds to the duration of the time interval reproduced by the subject minus the duration of the time interval set by the metronome and provides a direct measure of the magnitude of the error in reproducing the corresponding time interval (in ms). The interval reproduction accuracy index is the ratio between the time interval reproduced by the subject and the time interval set by the metronome, and allows a comparison of performance at each time interval, independent of duration. This index provides also the directionality of the tapping performance, being >1 if the participant is behind the beat and <1 if the participant is ahead of the beat. The temporal error and the interval reproduction accuracy index were analyzed to explore: (1) performance on the single-hand task with the right hand, (2) movement lateralization (comparison of right hand performance on single-hand and bimanual tasks), (3) bimanual coordination (comparison of right- and left-hand performance during the bimanual task).

### Statistical Analysis of Motor Performance

#### Single-Hand Task With the Right Hand

Temporal error and interval reproduction accuracy index were analyzed by means of a repeated measures analysis of variance (RM-ANOVA) with GROUP (TS patients, healthy subjects) as between-subjects factor and MODE (SYNC and CONT) and TIME INTERVAL (500 and 2,000 ms) as within-subjects factors.

#### Movement Lateralization

Temporal error and interval reproduction accuracy index were analyzed by means of a repeated measures analysis of variance (RM-ANOVA) with GROUP (TS patients, healthy subjects) as between-subjects factor and MODE (SYNC and CONT), TASK (single-hand, bimanual), and TIME INTERVAL (500 and 2,000 ms) as within-subjects factors.

#### Bimanual Coordination

Temporal error and interval reproduction accuracy index were analyzed by means of a repeated measures analysis of variance (RM-ANOVA) with GROUP (TS patients, healthy subjects) as between-subjects factor and MODE (SYNC and CONT), SIDE (right, left), and TIME INTERVAL (500 and 2,000 ms) as within-subjects factors.

We performed *post hoc* analyses of significant interactions using *t*-tests applying the Bonferroni correction for multiple comparisons where necessary. We considered *p* values lower than 0.05 as threshold for statistical significance. We performed statistical analysis with SPSS 13.0.

### Magnetic Resonance Imaging (MRI) Tractography Studies

#### Image Acquisition

Thirteen TS patients and 13 of the 19 healthy subjects underwent MRI-based diffusion tensor imaging and probabilistic tractography of inter-hemispheric corpus callosum (CC) connections between supplementary motor areas (SMA) and probabilistic tractography of the SMA-putamen connection of the left hemisphere.

Images were acquired on a 3T Siemens Trio MRI scanner (body coil excitation, 12-channel receive phased-array head coil). Anatomical scans were acquired using sagittal 3D T1-weighted magnetization prepared rapid acquisition gradient echo. The characteristics of diffusion weighted scans were as follow: echo time (TE): 87 ms; repetition time (TR): 12 s; 65 slices; matrix: 128 × 128; voxel size: 2 × 2 × 2 mm^3^; partial Fourier factor: 6/8; grappa factor: 2; read bandwidth: 1,502 Hz/pixel; flip angle: 9°. Diffusion weighting was performed along 50 directions with a b-value of 1,000 s·mm^−2^. We also obtained a reference image with no diffusion weighting. We asked TS patients to suppress their tics during the acquisition in order to avoid movement artifacts.

#### Image Processing

We performed image pre-processing using the FSL toolbox from the FMRIB Software Library. We corrected diffusion images for eddy current artifacts, and generated fractional anisotropy (FA) maps using FDT (FMRIB's Diffusion Toolbox). We applied the analytical Q-ball model to estimate the local underlying orientation distribution function (ODF) using a spherical harmonics order 6 and a regularization factor equal to 0.006 (21). We realigned the high-resolution 3D T1 volume to the diffusion data. We calculated the probabilistic distributions of the fiber orientations at each voxel using a constrained spherical deconvolution (CSD) model with the MRtrix software (22).

Probabilistic tractography was performed by the probtrackx toolbox of FSL software, using the left SMA a seed region of interest (ROI), and the left posterior putamen as waypoint and termination ROI. An exclusion mask was added on the midsagittal plane in order to avoid erratic fibers from the corpus callosum. ROI masks were created in each participant's diffusion space. The supplementary motor area (SMA) was anatomically defined as the medial cortex caudal to the VCA line of Talairach (line drawn through the anterior commissure perpendicular to the anterior commissure-posterior commissure line) (23). The posterior putamen was defined as the segment of the putamen caudal to the VCA line (24). The following parameters were used: 5,000 samples, curvature threshold 0.2. The tract mask from the probabilistic tractography map of each subject was used to compute the mean of FA weighted by track probability. We reconstructed tracts between the two SMA (SMA-SMA tract) regions and between the SMA and putamen for the left hemisphere. FA was extracted from each tract of interest in every subject.

#### Statistical Analysis: Neuroimaging Data

Mean FA measures from SMA-SMA and from left-sided SMA-putamen fiber tracts were compared between the two groups using univariate ANOVA (separately for each tract) with age as a covariate of non-interest.

We also performed correlation analyses between FA values of SMA-SMA and left-sided SMA-putamen fiber tracts and severity of tics measured by YGTSS severity sub-score and relevant outcome measures of the motor task using Pearson's correlation test. These correlation analyses were performed separately for the TS patients and HC groups. All statistical analyses were performed with SPSS 22.0.

## Results

### Single-Hand Task

#### Temporal Error

**Figure 2:**
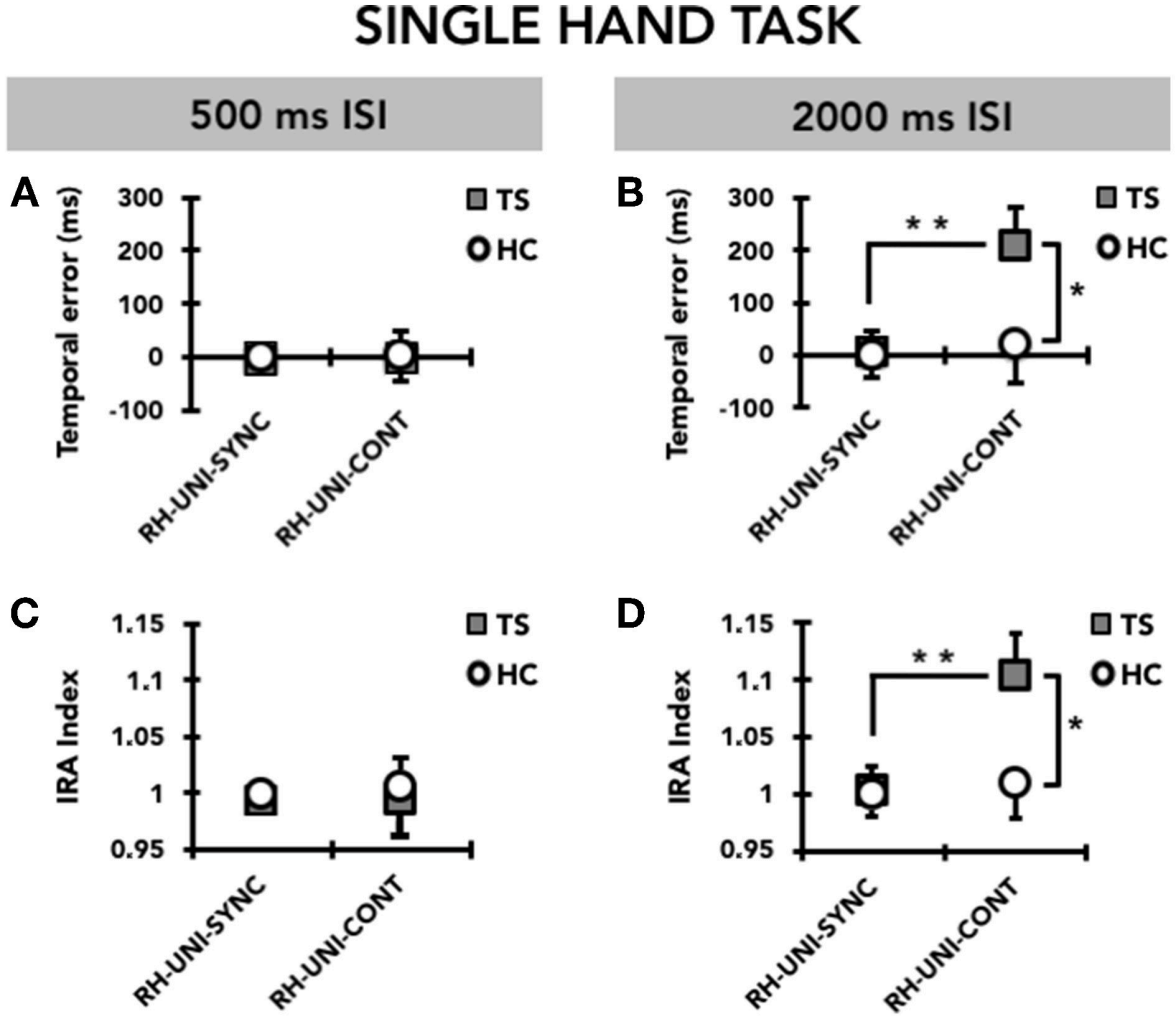
Timing performance on the single-hand task (UNI) performed with the right hand (RH). Data of both patients with Tourette syndrome (TS) and healthy control subjects (HC) are shown. The results of the synchronization (SYNC) and continuation (CONT) tasks with a supra-second (0.5 Hz, 2,000 ms ISI) and sub-second (2 Hz, 500 ms ISI) time interval are shown. On the x-axis, we show the type of task. On the y-axis, we show the duration of the temporal error in ms **(A,B)** and the IRA expressed as a ratio between the time interval reproduced by the subject and the time interval set by the metronome **(C,D)**. Asterisks indicate when statistical analysis showed a significant difference (*p < 0.05, **p < 0.01). Mean data + standard error mean (SEM) are shown.

RM-ANOVA showed a significant effect of the TIME INTERVAL^*^GROUP^*^MODE interaction term (F_(1, 31)_ = 4.20; *p* = 0.049). *Post hoc* analyses revealed that the temporal error for the supra-second time interval (2,000 ms, 0.5 Hz) was significantly larger in TS patients than in HC only in the CONT mode (*p* = 0.045), but not in the SYNC mode (*p* = 0.47 (Figure 2). Moreover, for the supra-second time interval, the temporal error was significantly larger in the CONT mode compared to the SYNC mode (*p* = 0.008) only in TS patients, but not in HC (*p* = 0.77). Finally, in TS patients only, temporal error in the CONT mode was significantly larger for the supra-second time interval compared to the sub-second time interval (TS, *p* = 0.005; HC, *p* = 0.98). *Post hoc* analyses revealed no difference in temporal error between TS patients and HC subjects for the sub-second time interval, either in the CONT or in the SYNC modes (all *p* > 0.05).

#### Interval Reproduction Accuracy Index

Like for temporal error, RM-ANOVA showed a significant effect of the TIME INTERVAL^*^GROUP^*^MODE interaction (F_(1, 31)_ = 4.42; *p* = 0.044) also for the interval reproduction accuracy index. *Post hoc* analyses revealed that the accuracy in reproducing the supra-second time interval (2,000 ms, 0.5 Hz) was reduced in patients with TS compared to HC only in the CONT mode (*p* = 0.045), but not in the SYNC mode (*p* = 0.48) (Figure 2). Whereas the interval reproduction accuracy for the supra-second time interval was similar between CONT and SYNC in HC (*p* = 0.77), this parameter was significantly larger (i.e., performance was less accurate) in CONT with respect to SYNC (*p* = 0.008) in TS patients, but not in HC (*p* = 0.78). This indicates that TS patients manifest a significant tendency to remain “behind the beat” when asked to reproduce the supra-second time interval without the metronome cueing. Finally, only in the CONT mode and only in TS patients, the reproduction accuracy for the supra-second time interval was lower than for the sub-second time interval (TS, *p* = 0.007; HC, *p* = 0.71). We did not find any difference in the reproduction accuracy for the sub-second time interval between TS patients and HC (all *p* > 0.05).

### Movement Lateralization

#### Temporal Error

**Figure 3:**
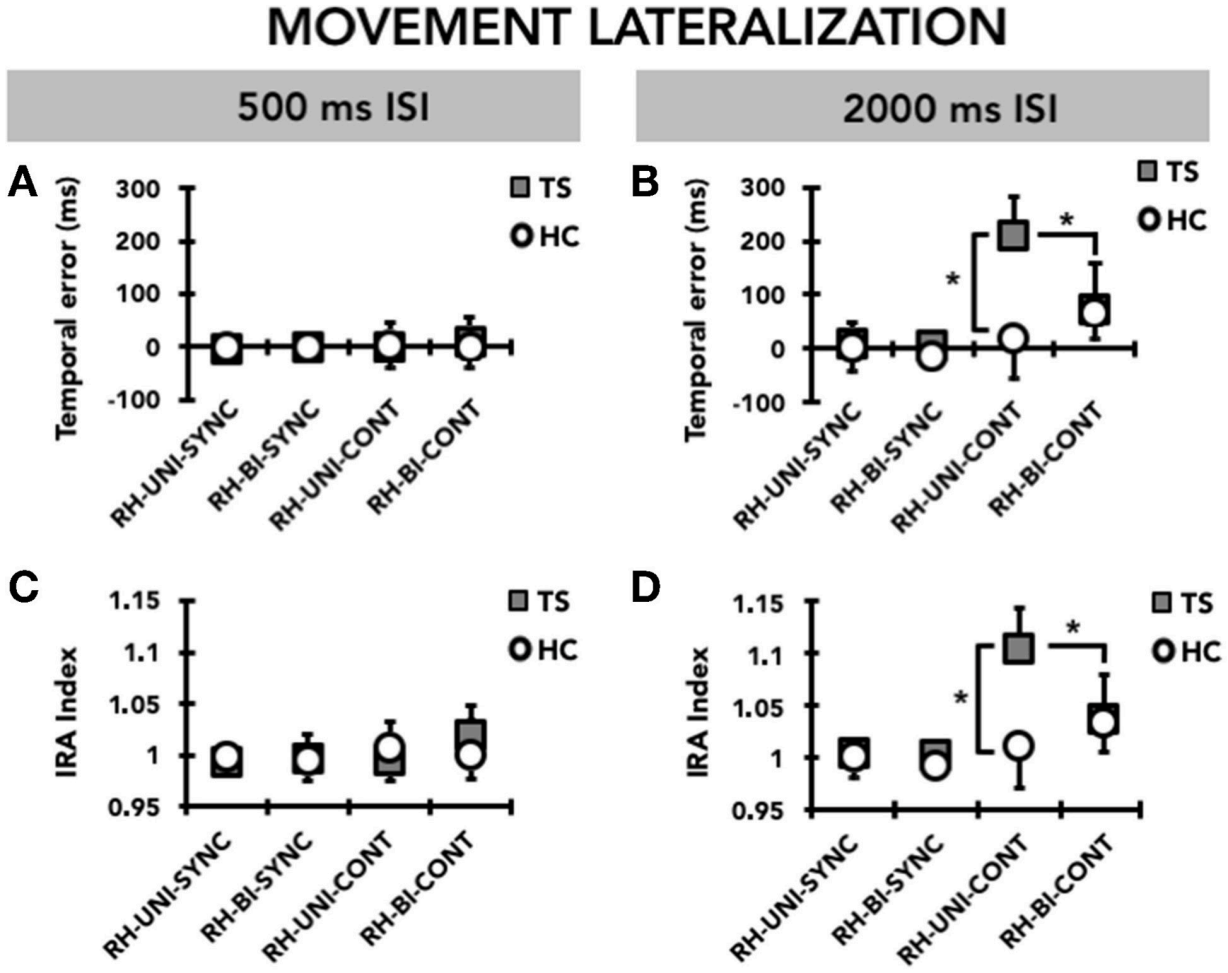
Movement lateralization: timing performance of the right hand (RH) on the single-hand task (UNI) and on the bimanual task (BI). Data of both patients with Tourette syndrome (TS) and healthy control subjects (HC) are shown. The results of the synchronization (SYNC) and continuation (CONT) tasks with a supra-second (0.5 Hz, 2,000 ms ISI) and sub-second (2 Hz, 500 ms ISI) time interval are shown. On the x-axis, we show the type of task. On the y-axis, we show the duration of the temporal error in ms **(A,B)** and the IRA expressed as a ratio between the time interval reproduced by the subject and the time interval set by the metronome **(C,D)**. Asterisks indicate when statistical analysis showed a significant difference (*p < 0.05). Mean data + standard error mean (SEM) are shown.

When we compared the timing ability of the right hand between the single-hand and the bimanual versions of the task, we observed that TS patients had greater timing ability on bimanual compared to single-hand (Figure 3). RM-ANOVA showed a significant effect of the TASK^*^MODE^*^TIME INTERVAL^*^ GROUP interaction term (F_(1, 31)_ = 7.97; *p* = 0.008). *Post hoc* analyses revealed that the temporal error was significantly larger in TS patients than in HC only for the supra-second time interval (2,000 ms, 0.5 Hz), on the single-hand version of the task and in the CONT mode (*p* = 0.048), but not on the bimanual version of the task or in the SYNC mode (all *p* > 0.05). Moreover, the temporal error of the right hand was significantly larger when reproducing a supra-second time interval compared to a sub-second time interval only in the single-hand version of the task, only in the CONT mode, and only in TS patients (*p* = 0.005). There was no statistically significant difference when comparing temporal error for supra-second and sub-second intervals in HC (all *p* > 0.05). Finally, for the supra-second time interval measured in the CONT mode, the temporal error of the right hand was significantly larger on the single-hand version of the task than on the bimanual version of the task in TS subjects (*p* = 0.03), but not in HC subjects (*p* = 0.34).

#### Interval Reproduction Accuracy Index

RM-ANOVA showed a significant interaction TASK^*^MODE^*^ TIME INTERVAL^*^ GROUP (F(1,31) = 10.34; *p* = 0.002) (Figure 3). *Post hoc* analyses revealed that reproduction accuracy for the supra-second time interval (2,000 ms, 0.5 Hz) was smaller in TS patients than in HC only on the single-hand version of the task and in the CONT mode (*p* = 0.045), but not on the bimanual version of the task and in the SYNC mode (all *p* > 0.05). Moreover, TS patients were less accurate when reproducing a supra-second time interval compared to a sub-second time interval with the right hand only in the single-hand version of the task and in the CONT mode (*p* = 0.007), whereas no difference emerged when comparing supra-second and sub-second timing performance in HC (all *p* > 0.05). Finally, reproduction accuracy for the supra-second time interval was smaller on the single-hand version of the task than on the bimanual version of the task only in TS subjects and in the CONT mode (*p* = 0.03), but not in the SYNC mode and in HC subjects (*p* = 0.34).

### Bimanual Coordination

#### Temporal Error

**Figure 4:**
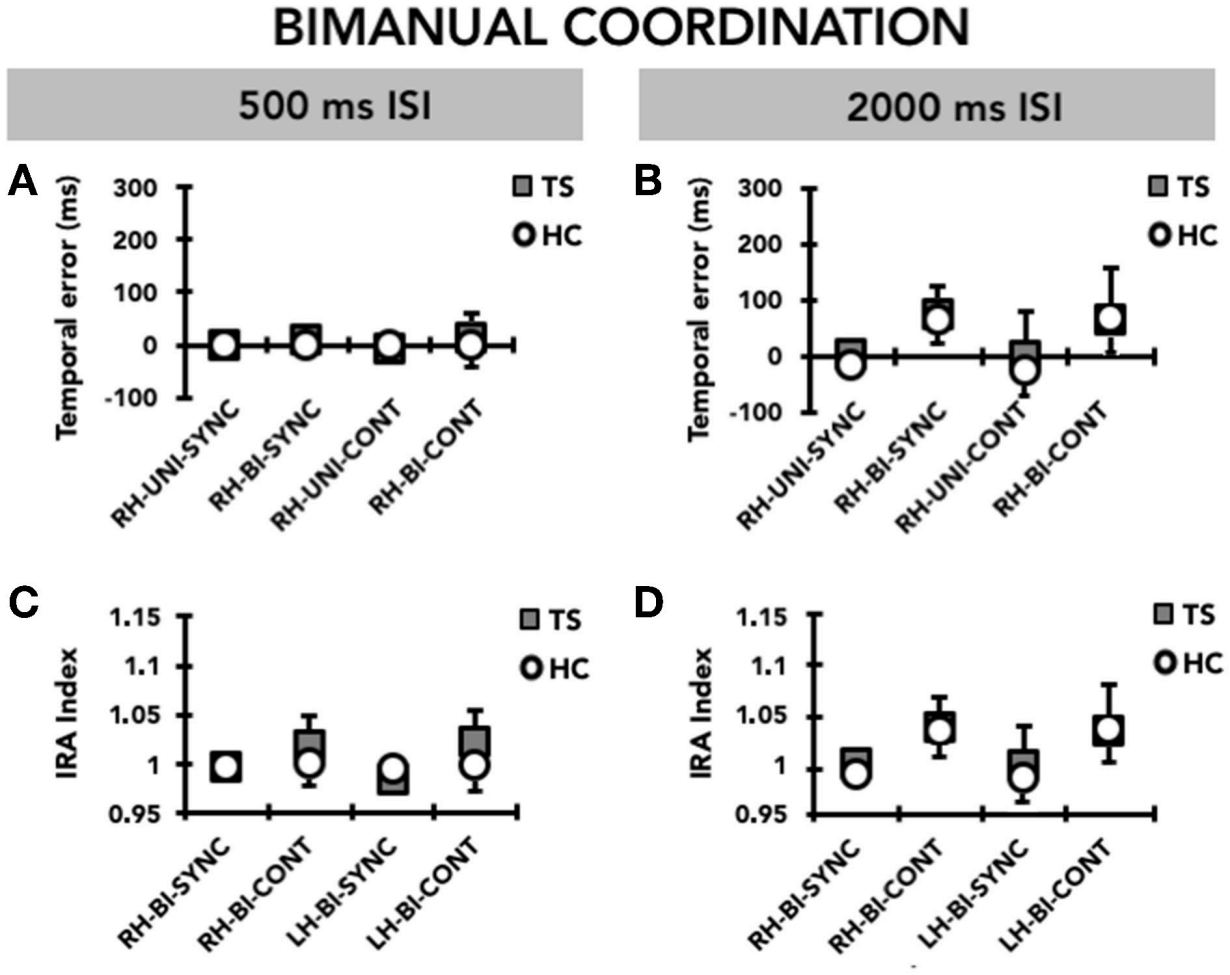
Bimanual coordination: timing performance of the right hand (RH) and the left hand (LH) on the bimanual task (BI). Data of both patients with Tourette syndrome (TS) and healthy control subjects (HC) are shown. The results of the synchronization (SYNC) and continuation (CONT) tasks with a supra-second (0.5 Hz, 2,000 ms ISI) and sub-second (2 Hz, 500 ms ISI) time interval are shown. On the x-axis, we show the type of task. On the y-axis, we show the duration of the temporal error in ms **(A,B)** and the IRA expressed as a ratio between the time interval reproduced by the subject and the time interval set by the metronome **(C,D)**. Mean data + standard error mean (SEM) are shown.

When we compared the timing ability of the right and left hand on the bimanual version of the task, we did not observe any difference either in the TS group or in the HC group (Figure 4). Accordingly, RM-ANOVA showed a significant effect only of MODE (F_(1, 31)_ = 4.42; *p* = 0.044); *post hoc* analysis showed a larger temporal error in the CONT compared to the SYNC mode (*p* = 0.044). We did not detect any other significant effect for any of the other factors, or interaction terms between GROUP and any of the within-subjects factors.

#### Interval Reproduction Accuracy Index

Similar to the temporal error, RM-ANOVA for interval reproduction accuracy showed a significant effect of MODE (F(1,31) = 5.36; *p* = 0.027), whereby performance in the CONT mode was less accurate than that recorded in the SYNC mode (*p* = 0.027). Again, we did neither observe a significant effect for any other main factor, nor a significant interaction between GROUP and any of the within-subjects factors, indicating that there was no difference between TS and HC (Figure 4).

### Tractographic Analysis

Compared to HC, TS patients yielded a significantly higher FA in both the SMA-SMA transcallosal tract (F_(1, 26)_ = 6.375, *p* = 0.018; mean ± SD FA: 0.551 ± 0.109 in TS patients and 0.499 ± 0.018 in HC) and the left SMA-putamen tracts (F_(1, 26)_ = 4.47, *p* = 0.04; mean ± SD FA: 0.478 ± 0.11 in TS patients and 0.415 ± 0.01 in HC). Importantly, there was no difference in the ROI mask size between groups in either hemisphere (all *p* > 0.5).

### Correlation Analysis

We did not observe any significant correlation between FA values and severity of tics measured by YGTSS total severity score (*p* > 0.5).

**Figure 5:**
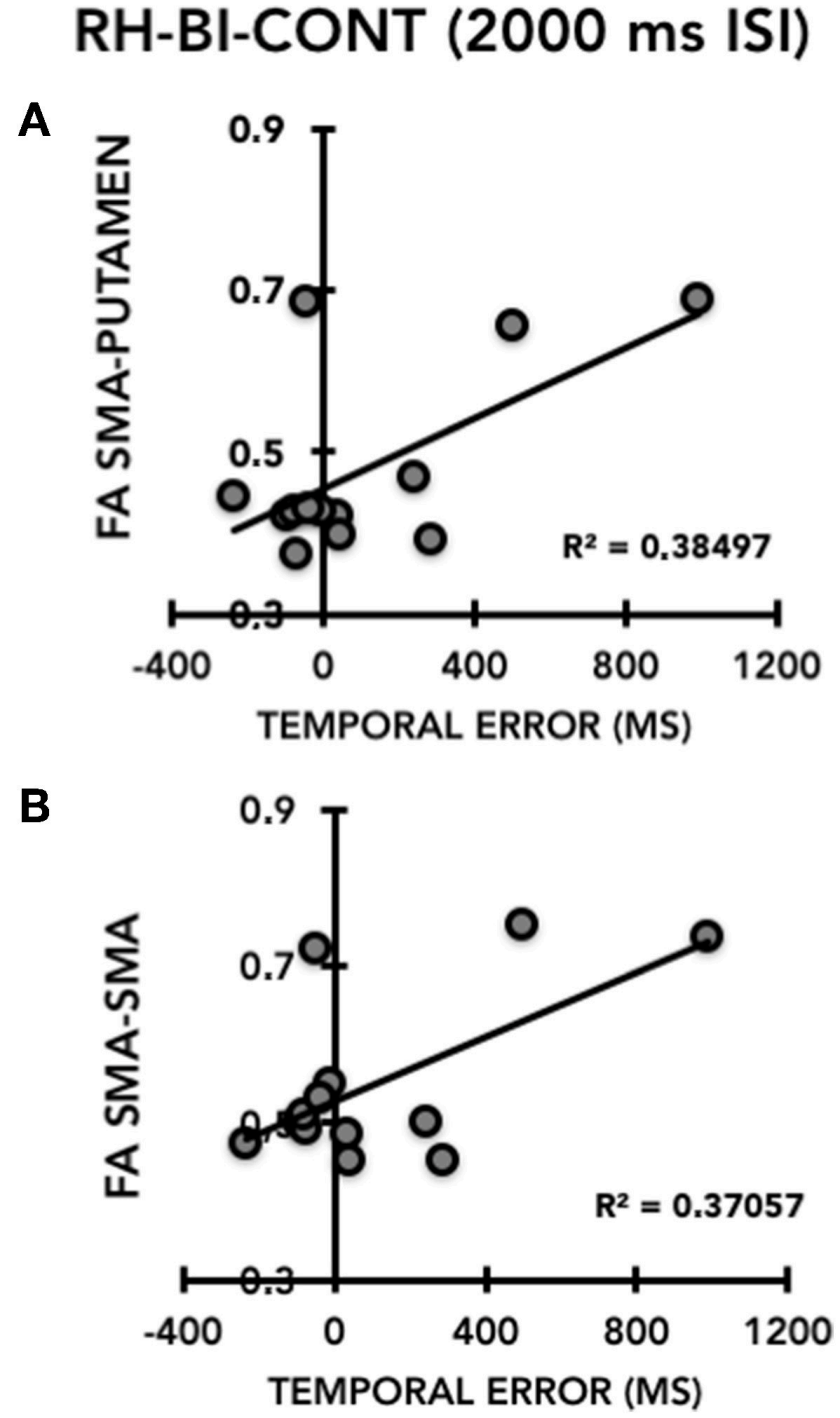
Correlation between the temporal error of the right hand (RH) on the bimanual task (BI), continuation task (CONT) in the supra-second time interval task (2,000 ms, 0.5 Hz) (X-axis) and the FA of the left SMA-putamen (Y-axis) **(A)** and the FA SMA-SMA tracts (Y-axis) **(B)** in patients with Gilles de la Tourette syndrome (TS).

We conducted correlation analyses in HC and TS patients separately between FA values and two selected motor outcome measures: (i) right hand temporal error for supra-second intervals in the CONT mode on the single-hand version of the task, selected because significantly different between TS patients and HC; (ii) right hand temporal error for supra-second intervals in the CONT mode on the bimanual version of the task, selected as a measure of movement lateralization. We chose not to conduct correlation analyses on interval reproduction accuracy because it yielded very similar between-group differences to temporal error and due to our limited sample size. There was no significant correlation in either the TS group or the HC group between FA values of the left SMA-putamen or of the SMA-SMA transcallosal tracts and right hand timing performance on the single-hand version of the motor timing task (all *p* > 0.5). Conversely, in TS patients we detected a positive correlation between the temporal error of the right hand on the bimanual version of the task in the CONT mode and for the supra-second time interval (2,000 ms, 0.5 Hz), and the FA value of the SMA-SMA tract (*r* = 0.60), which was just above the Bonferroni-corrected 0.025 (= 0.05/2) threshold of significance (*p* = 0.027 (Figure 5). The same motor outcome measure yielded a significant positive correlation with the FA value of the left SMA-putamen tract (*r* = 0.62, *p* = 0.024) of TS patients. The same correlation analysis was not significant for HC (all *p* > 0.05 (Figure 5). Hence, in our TS patients we observed that the worse the timing performance of the right hand for the supra-second time interval on the bimanual version of the task (CONT mode only), the higher the FA value in the SMA-SMA and left SMA-putamen tracts.

## Discussion

In the present study, we adopted a synchronization-continuation task to investigate motor timing abilities in adult patients with TS and age-matched healthy volunteers. Further, we compared the performance of the right hand between a single-hand and a bimanual version of the same motor timing task to assess movement lateralization. Finally, we compared the performance of the two hands on the bimanual version of the task to assess bimanual coordination. Our task is an explicit motor timing task, in which the goal is to provide an accurate estimate of time intervals (4). During the metronome-cued phase (SYNC mode), subjects stored temporal information related to an auditory stimulus presented at regular inter-stimulus intervals (ISI) whilst reproducing the same ISI synchronously with the cue. During the non-cued phase (CONT mode), subjects used the temporal information acquired during the previous phase to continue reproducing through a motor sequence the same ISI, but in the absence of the auditory pacing cue.

In line with our hypotheses, we observed that adults with TS exhibit a specific deficit of motor timing accuracy with the right (dominant) hand, in which they consistently remain “behind the beat” when asked to reproduce a supra-second interval of 2,000 ms in the absence of metronome cueing (CONT mode). The reproduction accuracy is similar to healthy volunteers when patients reproduce the same interval synchronously with the metronome, or when they reproduce a sub-second interval of 500 ms.

The present findings add to a very scarce body of evidence of general and motor timing abilities in TS. Earlier studies (25, 26) failed to detect abnormalities of time estimation and reproduction in TS adults, but their recording methodology (a manual stopwatch) was inaccurate. Subsequently, using computerized timing tasks (27), our group showed that a pediatric TS population was more accurate than age-matched youth in the reproduction of supra-second time intervals using a motor action (tapping a keyboard key) to signal the end of the interval. In that study, timing accuracy correlated negatively with tic severity, which led to speculate a link between enhanced ability in time processing and adaptive changes within prefronto-basal ganglia circuits related to inhibitory control over tics. In a subsequent work, we reported that dopaminergic modulation with dopamine receptor blockers could improve the variability on a temporal discrimination task in TS children (28).

While these previous studies explored only the perceptual aspects of timing, the present study is the first in TS to address timing accuracy in the context of a fine motor task. The neural substrate of the motor performance exhibited during the synchronization-continuation task spans across the sensorimotor loop of the cortico-basal ganglia circuitry (interconnecting the dorsolateral striatum, the ventral thalamus, and primary motor and somatosensory, premotor and supplementary motor cortical regions) and cortico-cerebellar circuits. In particular, the striatal dopaminergic tone is believed to modulate a hypothetical “internal clock” beating the rhythm during internally generated movements. A recent conceptualization of temporal processing (16) suggests a unified timing mechanism across both sub- and supra-second intervals. According to this model, different regions within the deeply interconnected network involving both cortico-basal ganglia and cerebellar output pathways may specialize in different phases of time processing, albeit working in conjunction and across the whole spectrum of temporal durations. A body of evidence supports a prominent involvement of cerebellar output pathways in the initiation and adjustment of timing when facing a novel timing task (29–31), whereas the cortico-striato-thalamo-cortical circuits are more involved in the continuation phase or in the initiation of previously learned temporal durations (16, 32, 33). Following this conceptualization of timing, the synchronization-continuation task appears to be an ideal paradigm to inform on the neural substrate underlying selective deficits in motor timing abilities, such as that manifested by TS patients in this study. Along these lines, the specific decrease in motor timing accuracy in the continuation mode and for a supra-second interval is suggestive of a partial deficiency to sustain the accuracy of explicit motor timing, a functional domain likely to be subserved by the cortico-striato-thalamo-cortical circuit (34), in particular by its sensorimotor loop. In keeping with this, paced finger tapping tasks at supra-second intervals were found to be executed at a lower degree of accuracy in patients with different basal ganglia disorders, and this was associated with reduced activation within primary sensorimotor and supplementary motor cortical regions on functional imaging (4, 35–37). The lack of correlation between the timing inaccuracy on the single hand version of our task and tic severity does not confirm our a priori hypothesis, and suggests that explicit motor timing deficiencies do not directly reflect neural mechanisms underlying tic generation, but more likely represent a “trait” marker of dysfunction in this putative explicit timing network.

The second part of our findings indicates, in keeping with our a priori hypothesis, that the selective motor timing abnormality expressed, on a group basis, by the right hand of TS patients on the single-hand version of the task is no longer manifest when the same task is simultaneously performed by both hands. This suggests that the implementation of a bimanual set-up of a novel, moderately skilful manual task activates adaptive mechanisms that counteract this timing inaccuracy and/or that bimanual execution facilitates motor timing performance.

Previous studies from our group (15, 38) have demonstrated that TS patients of different age groups are more accurate in executing bimanually than single-handedly a fine manual task like the finger opposition task used herein. In our previous work on the same sample of TS adult patients, we observed that the gain in accuracy observed on the bimanual task compared to the single-hand one was larger when tics were less severe, suggesting this is likely an epiphenomenon of compensatory mechanisms (15). Furthermore, when we explored the neural substrate of the lateralization performed expressed as percentage of spatially correct sequences (15), we found that TS patients (i) exhibit higher structural organization of transcallosal connections between the primary motor and supplementary motor area and that (ii) they had lost the physiological association between the ability to lateralize motor performance and the transcallosal connectivity of these motor cortical regions. We therefore concluded that the abnormality to lateralize finger movements in the sequential tasks in TS could be the effect of neural compensation involving the transcallosal pathway with the aim to self-regulate motor control.

Whereas, these previous studies measured task accuracy as the percentage of correct movements, thus focusing on spatial structural aspects of the motor sequence, the present study explores the degree of lateralization of explicit motor timing abilities. In the present study, the same TS sample of adult patients used for the previous study exhibited higher structural organization of transcallosal connections between the supplementary motor regions and of the left SMA-putamen, confirming findings from our previous work (15). Furthermore, when the motor timing accuracy of the right hand on the bimanual version of the task was lower (i.e., larger temporal error), the structural organization of transcallosal and subcortical connections of left SMA was higher. Although based only on cross-sectional observation, this increase of the ipsi- and contralateral connectivity of a key nodal region in the cortico-striato-thalamo-cortical circuitry like the SMA can be interpreted as the consequence of an attempt to compensate for a functionally broader (i.e., both single hand and bimanual) deficit in motor timing accuracy. Consistently, Buse et al. (39) reported that the callosal sub-region 3, which includes the fiber tracts examined in our study, exhibits progressive growth over time during development in TS patients, probably underlying an attempt to accelerate interhemispheric transfer as a compensatory process. However, in the present study we did not observe any significant correlation between neither timing performance in the single-hand nor in the bimanual version of the task and severity of tics. A possible explanation relies on the nature of the task that, by exploring motor timing abilities, is more dependent on a larger cortico-subcortical (involving also the prefrontal cortex) network than a pure motor task reliant only on accuracy of finger movements. Thus, even if there is not a direct link between motor timing ability and tic expression (differently from accuracy in motor performance).

Overall we interpret our cumulative evidence as an abnormality to lateralize finger movements in sequential tasks occurring in TS as a consequence of compensatory mechanisms in neural organization.

In conclusion, our study demonstrates that TS patients manifest “trait” abnormalities in the timing of sequential motor tasks, which are in keeping with the continuation phase of time processing, likely controlled by the sensorimotor loop of the cortico-basal ganglia network. We also show that the abnormal lateralization of fine motor control, previously reported in the context of the structural sequencing of fine motor tasks, extends also to motor timing accuracy. Finally, we highlight SMA connectivity as a potentially pivotal neural substrate of adaptive compensation of motor timing deficits in fine manual tasks in TS. We acknowledge that our results are based on a relatively small sample size. Another potential limitation is the lack of assessment for sub-diagnostic threshold ADHD symptomatology, although none of our TS patients had any history of current or past ADHD diagnosis. In this respect, future studies on larger samples should explore the presence of compensatory activation patterns using functional MRI, and correlate the level of this compensatory activation to the pattern of lateralization of motor timing abilities in TS patients. As for many other aspects of this complex neurodevelopmental disorder, longitudinal studies of multivariate datasets combining brain structure, brain performance and brain activation would have the potential to reveal the temporal trajectory of compensatory mechanisms that underlie phenotypic heterogeneity.

## Ethics Statement

The local ethics committee (Pitie -Salpêtrière Hospital) approved the study and every participant gave informed written consent for participation. The Ethics committee project' number is INSERM C11-34, CPP 97/12.

## Author Contributions

LA, DM, YW, and AH conceived and designed the experiments and interpreted the data and critically revised the article for important intellectual content. LA, EP, CD, and DM performed the experiments. EP, GL, CD, and YW analyzed the data. LA, DM, EP, GL, CD, YW, and AH wrote the paper. LA, DM, and EP drafted the article.

### Conflict of Interest Statement

The authors declare that the research was conducted in the absence of any commercial or financial relationships that could be construed as a potential conflict of interest.
